# Iron Reduction in *Dermacentor andersoni* Tick Cells Inhibits *Anaplasma marginale* Replication

**DOI:** 10.3390/ijms23073941

**Published:** 2022-04-01

**Authors:** Muna Salem M. Solyman, Jessica Ujczo, Kelly A. Brayton, Dana K. Shaw, David A. Schneider, Susan M. Noh

**Affiliations:** 1 Department of Veterinary Microbiology and Pathology, Washington State University, Pullman, WA 99164-7040, USA; muna.solyman@uob.edu.ly (M.S.M.S.); kbrayton@wsu.edu (K.A.B.); dana.shaw@wsu.edu (D.K.S.); 2 Animal Diseases Research Unit, United States Department of Agriculture, Agricultural Research Service, 3003 ADBF, Pullman, WA 99164-6630, USA; jessie.ujczo@gmail.com (J.U.); david.schneider1@usda.gov (D.A.S.)

**Keywords:** *Anaplasma marginale*, iron transport, *Dermacentor andersoni*, tick-borne disease, siderophore-independent iron transport, anaplasmosis, obligate intracellular bacteria

## Abstract

*Anaplasma* spp. are obligate intracellular, tick-borne, bacterial pathogens that cause bovine and human anaplasmosis. We lack tools to prevent these diseases in part due to major knowledge gaps in our fundamental understanding of the tick–pathogen interface, including the requirement for and molecules involved in iron transport during tick colonization. We determine that iron is required for the pathogen *Anaplasma marginale*, which causes bovine anaplasmosis, to replicate in *Dermacentor andersoni* tick cells. Using bioinformatics and protein modeling, we identified three orthologs of the Gram-negative siderophore-independent iron uptake system, FbpABC. Am069, the *A. marginale* ortholog of FbpA, lacks predicted iron-binding residues according to the NCBI conserved domain database. However, according to protein modeling, the best structural orthologs of Am069 are iron transport proteins from Cyanobacteria and *Campylobacter* *jejuni*. We then determined that all three *A. marginale* genes are modestly differentially expressed in response to altered host cell iron levels, despite the lack of a Ferric uptake regulator or operon structure. This work is foundational for building a mechanistic understanding of iron uptake, which could lead to interventions to prevent bovine and human anaplasmosis.

## 1. Introduction

The family *Anaplasmataceae* includes bacterial pathogens that cause disease in humans and animals. These pathogens include *Anaplasma marginale* and *A. phagocytophilum*, which cause bovine and granulocytic anaplasmosis, respectively. The Ehrlichial species *Ehrlichia ruminantium*, *E. chaffeensis*, and *E. canis* cause ‘heartwater’ in ruminants and monocytic ehrlichiosis in humans and dogs, respectively. Overall, effective measures to prevent these diseases are lacking. The fact that these pathogens are all tick transmitted presents opportunities to develop novel, broadly applicable interventions to prevent disease. However, major fundamental knowledge gaps about the molecular interactions between the tick and the pathogen that allow for successful transmission of the pathogen limit our ability to develop such interventions. These interventions could include antibodies or other immune effectors produced by vaccination of the host and delivered during tick feeding or novel chemical inhibitors.

With few exceptions, iron is an essential and limiting nutrient for bacterial pathogens and their hosts. It is a critical component of eukaryotic and prokaryotic cellular functions and is indispensable for nucleic acid and lipid synthesis, protein translation, and energy metabolism and generation [1,2]. Because iron is highly reactive and essential, it is tightly regulated in eukaryotic systems, and bacterial pathogens have developed varied molecules and mechanisms for obtaining iron from the eukaryotic host. Thus, methods that block the ability of the pathogen to acquire iron may serve as the foundation of intervention strategies.

However, we know very little about the requirement for, or the molecules and mechanisms that mediate iron uptake in *Anaplasma* and *Ehrlichia* spp. during tick colonization. On the basis of the presence of a heme biosynthesis pathway, *Anaplasma* and *Ehrlichia* spp. are expected to require iron. Deferoxamine, an iron chelator, nearly completely abrogates *E. chaffeensis* and partially reduces *A. phagocytophilum* infection in monocytes [3]. A robust understanding of iron uptake and metabolism in macrophages has served as the foundation for understanding how *E. chaffeensis* and other pathogens acquire iron from host cells [4,5].

Relative to macrophages and other mammalian cells, iron uptake and metabolism in ticks is poorly understood. Because ticks lack heme oxygenase and thus do not acquire iron from heme, it has been proposed that ticks may acquire iron from mammalian transferrin in the blood meal [6]. However, ticks apparently lack transferrin receptors (TfR), and the mechanisms and molecules used to acquire iron from the blood meal are unknown. Additionally, the mechanisms of iron regulation are poorly understood. For example, in vertebrates, the iron-responsive protein (IRP) and iron-responsive elements (IREs) interact to form a regulatory system that controls translation of many genes involved in iron metabolism, including ferritins that store cytosolic iron, TfR1, and divalent metal transporter 1 (DMT1), among others. In contrast, ferritin 1 is the only gene in ticks with an IRE regulating the translation of ferritin 1, which stores cytosolic iron in the midgut [6]. These differences in iron uptake and metabolism in tick cells as compared to mammalian cells suggest the requirement and strategies used by the pathogen may vary depending on the species of the host and type of host cell.

Based on bioinformatics, there are few identifiable genes or iron-dependent regulators directly involved in iron uptake in *Anaplasma* and *Ehrlichia* spp. [7,8]. These pathogens apparently lack iron-dependent repressors, iron response regulators, heme and transferrin receptors, heme oxygenase required to liberate iron from heme, and siderophores. However, three genes are predicted to be involved in iron uptake and are conserved among *Ehrlichia* and *Anaplasma* spp. In *A. marginale*, these genes are *Am069*, *Am240*, and *Am392* (locus identifiers based on the St. Maries strain, RefSeq GCF_000011945.1). The proteins encoded by these genes are orthologs of the siderophore-independent iron uptake systems first defined in *Neisseria* spp. (nFbpABC) and *Haemophilus influenzae* (hFbpABC) and found in many other Gram-negative pathogens [9,10,11]. Typically, these genes are encoded on an operon and are under the negative regulatory control of the ferric uptake regulator (*fur*) [10,12,13]. However, the *Anaplasmataceae* lack a *fur* regulator, and these genes are dispersed throughout the genome rather than being encoded in an operon. There are no data regarding a possible role of these genes in iron transport.

In this study, we used *A. marginale*, which is transmitted by *Dermacentor* spp. ticks, to address these knowledge gaps by testing two hypotheses: (1) iron is required for *A. marginale* replication in *D. andersoni* cells; (2) the FbpA, FbpB, and FbpC orthologs in *A. marginale* are differentially expressed in response to iron deprivation during tick cell colonization. The findings are discussed and reviewed in the context of knowledge gaps in our understanding of iron acquisition by obligate intracellular, tick-borne bacterial pathogens.

## 2. Results

### 2.1. Bpdl Had a Minimal Effect on DAE100 Cell Viability

**Figure 1:**
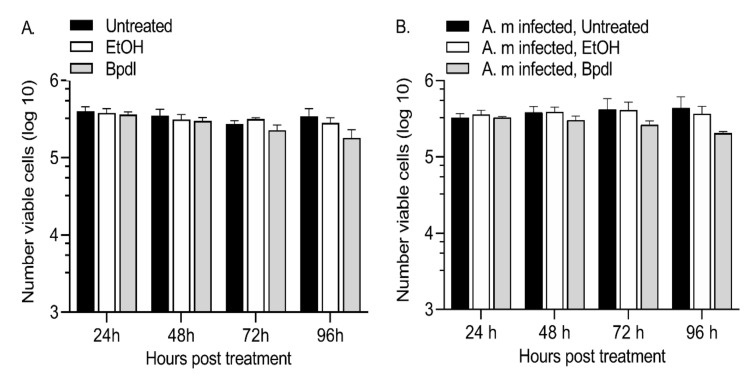
Minimal cell death in response to Bpdl treatment. There were minimal differences in the number of live cells following Bpdl treatment in uninfected (**A**) and *A. marginale*-infected (**B**) DAE100 cells. DAE100 cells received L15B complete medium (untreated), the carrier (EtOH), or 100 μM Bpdl. The number of viable cells was measured using trypan blue staining with three independent replicates. The bars represent the average ± standard error of the mean (SEM).

2,2′Bipyridyl (Bpdl), an iron chelator, has been successfully used to deplete *D. andersoni* cells (DAE100) of iron using a protocol similar to that used in these experiments [14]. First, we measured the cytotoxic effect of this chelator and its carrier, EtOH, through time to confirm that this treatment has minimal effects on uninfected and *A. marginale*-infected DAE100 cells (Figure 1, Table S1). There was little difference in the number of live DAE100 cells treated with the chelator compared to DAE100 cells receiving medium or the carrier at 24 h, 48 h, and 72 h of treatment. At 96 h of treatment, there was some cell loss in the group treated with the chelator, which was more prominent in the *A. marginale*-infected cells but was not statistically significant.

### 2.2. Anaplasma Marginale Levels Were Decreased in Response to Iron Depletion

**Figure 2:**
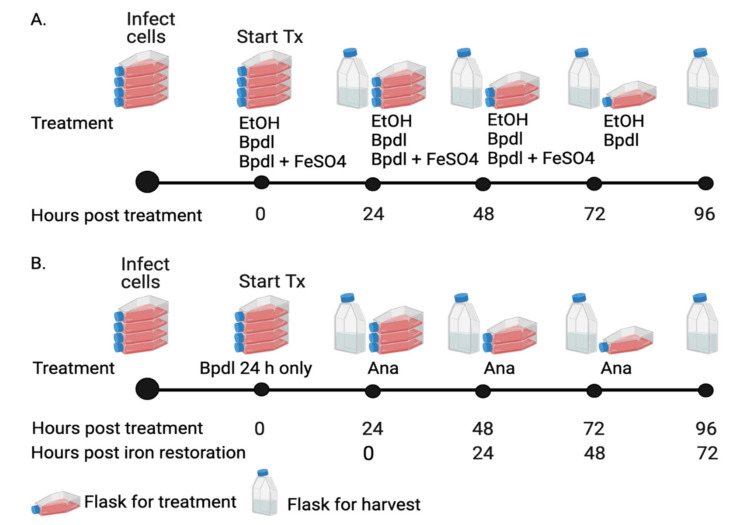
Experimental design. Timeline for *A. marginale* infection, treatments, and cell harvests for (**A**) iron depletion using Bpdl and recovery with FeSO_4_ in DAE100 cells and (**B**) reversal of iron depletion using iron replete, *Anaplasma* (Ana) medium. For all experiments, cells were infected with *A. marginale* 24 h prior to the initiation of treatments. (**A**) DAE100 cells were treated every 24 h with Bpdl, the carrier ethanol (EtOH), or Bpdl plus various concentrations of FeSO_4_. Cells were harvested starting 24 h following the first treatment and each 24 h thereafter. Cells treated with Bpdl or the carrier were harvested up to 96 h post-treatment, while cells treated with Bpdl plus FeSO_4_, were harvest up to 72 h post-treatment. (**B**) To reverse iron depletion, *A. marginale*-infected DAE100 cells were treated with Bpdl for 24 h only. Cells received *Anaplasma* medium for the remainder of the experiment. Cells were harvested starting 24 h following the first treatment and each 24 h thereafter up to 96 h post-treatment, which corresponded to 72 h after iron restoration.

**Figure 3:**
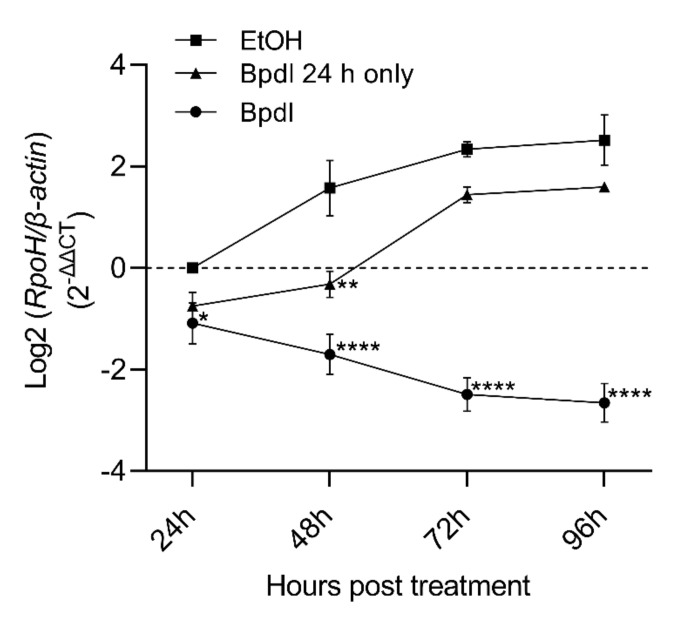
*Anaplasma marginale* levels in response to iron depletion and reversal of iron depletion. *Anaplasma marginale* levels in DAE100 cells maintained with the iron chelator Bpdl through time decreased as compared to the carrier control group (EtOH). *Anaplasma marginale* levels partially recovered following removal of the chelator after 24 h (Bpdl 24 h only). The y-axis is the log2 of the relative normalized expression of *A. marginale rpoH* relative to *D. andersoni ß-actin*. The average ± SEM of four independent replicates for Bpdl and three independent replicates for Bpdl 24 h only treatment is represented by the nodes and error bars. Asterisks indicate statistically significant differences between the Bpdl-treated, or recovered cells as compared to the carrier control at each time point. Statistical significance is denoted as follows: * = *p* < 0.05, ** = *p* < 0.01, **** = *p* < 0.0001.

In order to test the effect of iron depletion on *A. marginale* replication, DAE100 cells were infected with *A. marginale* for 24 h and then treated every 24 h with the iron chelator or the carrier only (Figure 2A). The amount of *A. marginale* was then measured with real-time quantitative PCR (RT-qPCR) over time. In cells treated with the carrier, *A. marginale* levels increased an average of 3.6-fold at 48 h and 5.1- and 6.3-fold at 72 h and 96 h, respectively (Figure 3, Table S2). In contrast, in the iron-depleted cells, *A. marginale* levels decreased on average by 1.9- and 3.0-fold at 24 h and 48 h, respectively, and 5.2- and 5.9-fold at 72 h and 96 h, respectively, compared to the cells treated with the carrier only at 24 h after treatment. At 48 h, 72 h, and 96 h, *A. marginale* levels were significantly (*p* < 0.0001) lower in the cells treated with the chelator as compared to the cells treated with the carrier only.

### 2.3. Recovery of A. marginale Replication following Removal of Bpdl

To determine if *A. marginale* replication could be restored following iron depletion, *A. marginale*-infected cells were treated with the chelator for 24 h only, after which cells received iron-replete *Anaplasma* medium for the remainder of the experiment (Figure 2B). Overall, there was approximately 50% recovery of *A. marginale* following the removal of the chelator.

Twenty-four hours after restoring iron levels (Bpdl 24 h only; 48 h post treatment), there was minimal *A. marginale* replication, and the Bpdl-treated (*p* < 0.0001) and Bpdl 24 h only groups (*p* = 0.0011) were significantly different from the carrier control (Figure 3, Table S2). Between 24 h to 48 h following restoration of iron levels (48 h and 72 h post-treatment), there was marked *A. marginale* replication in the Bpdl 24 h only group. At 72 h and 96 h post-treatment, the only significant differences were between the carrier-treated cells and those that were treated with the chelator (*p* < 0.0001). Throughout the course of the experiment, *A. marginale* levels increased 6.3- and 3.04-fold, in the carrier control cells and Bpdl cells treated for only 24 h, respectively (Figure 3).

### 2.4. Iron Reduction Significantly Decreased A. marginale Colony Number and Size

**Figure 4:**
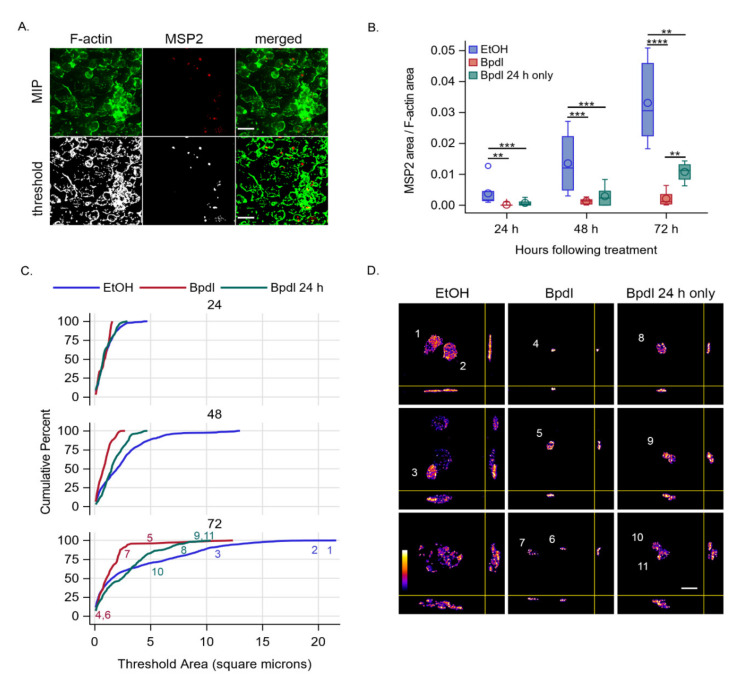
Bpdl inhibited intracellular growth of *A. marginale* in DAE100 cells as determined by immunofluorescence. (**A**) An example of deconvolved and segmented image stacks at 200× magnification showing DAE100 cells treated only with the carrier and infected with *A. marginale*. The top row are maximum intensity projections (MIP) of deconvolved images showing F-actin in DAE100 cells (green) and intracellular colonies of *A. marginale* (red) at 72 h post-infection. The second row (threshold) shows the corresponding z-axis projections after segmentation by automated local threshold analysis. The corresponding merged images are shown at the end of each row and include 30 micron scale bars, the approximate maximum profile width of DAE100 cells as visualized by F-actin. (**B**) A graph demonstrating the reversable inhibitory effects of Bpdl treatment on intracellular bacteria mass (total areas of segmented Msp2 divided by segmented F-actin). In comparison to treatment with the carrier alone (blue boxplots), treatment with Bpdl (red boxplots) significantly inhibited the growth of *A. marginale* colonies at all timepoints. In cells treated with Bpdl for 24 h only (green boxplots), intermediate recovery of growth was significant at 72 h. Significance markers: ** = *p* < 0.01, *** = *p* < 0.001, **** = *p* < 0.0001. (**C**) The reversible effects of Bpdl on growth of bacterial colonies as visualized by the empirical distribution functions of colony profiles (threshold areas). Note the proportional growth of intracellular bacterial colonies treated only with the carrier (EtOH; blue curves) into profiles greater than 5 and then 10 square microns, the near complete suppression of colony growth to profiles less than 5 square microns when continuously exposed to Bpdl (red curves), and the intermediate recovery of colony growth when only exposed to Bpdl in the first 24 h. Colored numbers in the graph at 72 h are positioned along the x-axis to indicate the approximate profile size of the corresponding three-dimensional examples shown in (**D**); deconvolved MIPs and orthogonal views at 630× magnification; scale bar in lower right panel = 5 microns; relative fluorescence intensity scale shown in bottom left panel).

Fluorescent imagining was used to estimate total intracellular bacterial mass through time. As depicted in Figure 4A, the use of local threshold algorithms on image stacks acquired at 200× magnification and sharpened by deconvolution resulted in well-segmented structures of DAE100 cells (F-actin marked by phalloidin-AlexaFluor488) and colonies of *A. marginale* (anti-Msp2 marked by AlexaFluor594). The intracellular bacterial mass was expressed in each image as the total area of threshold Msp2 divided by the total area of threshold phalloidin. The effects of treatment and incubation period on intracellular bacterial mass were significant (*p* < 0.0001). Treatment with Bpdl significantly reduced intracellular bacterial mass relative to the carrier-treated controls at all time points (Figure 4B). In cells treated with Bpdl for only 24 h, the intracellular bacterial mass partially recovered, and by 72 h was significantly greater than the cells treated with the chelator (*p* = 0.0014).

At 200× magnification, each discrete area of threshold Msp2 is an x–y-axes profile of the size of a bacterial colony. To assess the effects of treatment and time on colony size, the range and relative frequency of discrete threshold Msp2 areas from all imaged slide regions were visualized as empirical distribution functions (Figure 4C). At 24 h post-treatment, all imaged colonies were less than 5 square microns in profile and 80% or more were less than 2.5 square microns. The expansion of colony size over time in the carrier-treated cultures was readily apparent. Specifically, a substantial proportion of colony profiles were >5 square microns by 48 h incubation and greater than 10 square microns by 72 h post-treatment. In contrast, there was little evidence of colony growth in cells treated with the chelator, and most colony profiles remained at less than 5 square microns at 72 h post-treatment. When exposure to the chelator was limited to 24 h, expansion of colony size was apparent at 72 h, with a substantial proportion of colony profiles greater than 5 square microns. Examples showing the range of colony morphologies at 72 h post-treatment are shown at a higher resolution (630× magnification) in Figure 4D; the location of different profile sizes (numbered) are shown on the respective empirical distribution function for each treatment group (Figure 4C).

### 2.5. Addition of FeSO_4_ Restored A. marginale Replication in the Presence of Bpdl

**Figure 5:**
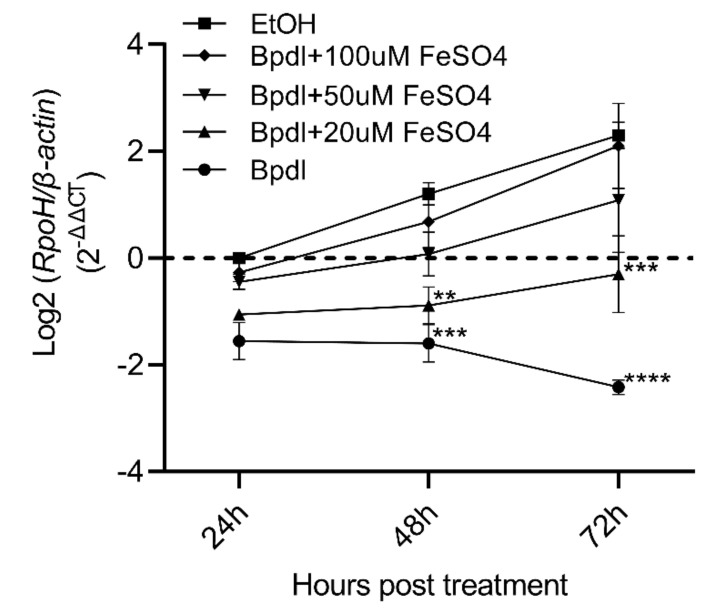
FeSO_4_ overwhelmed Bpdl chelation to restore *A. marginale* levels. There was a dose-dependent increase in *A. marginale* levels in DAE100 cells that received the iron chelator plus FeSO_4_ as compared to cells treated only with the chelator (Bpdl). The y-axis is the log2 of the relative normalized expression of *A. marginale rpoH* relative to *D. andersoni ß-actin*. The average ± SEM of four independent replicates is represented by the nodes and error bars. Asterisks indicate statistically significant differences between the carrier-treated cells (EtOH) and all other treatments at each time point. Statistical significance is denoted as follows: ** = *p* < 0.01, *** = *p* < 0.001, **** = *p* < 0.0001.

To confirm that the reduction in *A. marginale* levels was due to iron reduction in the host cell rather than unknown effects of the chelator, *A. marginale*-infected cells were treated every 24 h with *A. marginale* iron-free medium containing the chelator and 20 μM, 50 μM, or 100 μM of FeSO_4_ (Figure 5, Table S3). There was a dose-dependent increase in *A. marginale* levels in cells treated with the chelator and increasing amounts of FeSO_4_. There were no statistically significant differences between the cells treated with the carrier and those treated with the chelator plus 50 μM or 100 μM FeSO_4_ at all time points. The differences in *A. marginale* levels between the cells treated with the chelator only and the chelator plus 20 μM of Bpdl as compared to the carrier-treated cells were statistically different at 48 h (*p* < 0.01) and 72 h (*p* < 0001) post-treatment.

### 2.6. Predicted Function of Am069, Am240, and Am392

On the basis of a search of ABC transporters in the Kyoto Encyclopedia of Genes and Genomes (KEGG), there are three *A. marginale* genes predicted to be involved in iron transport: *Am069*, *Am240*, and *Am392*. The KEGG orthologs of these proteins are FbpA, FbpB, and FbpC, respectively. In general, FbpABC form a siderophore-independent iron transport system found in many extracellular and facultative intracellular, Gram-negative bacteria [11]. They are best defined in *Neisseria* spp. (nFbpABC) and *Haemophilus* spp. (hFbpABC) [9,10,15]. FbpA is involved in iron transport across the periplasm, while FbpB is a cytoplasmic permease, and FbpC, hydrolyzes nucleotide triphosphate to provide energy for transport.

**Figure 6:**
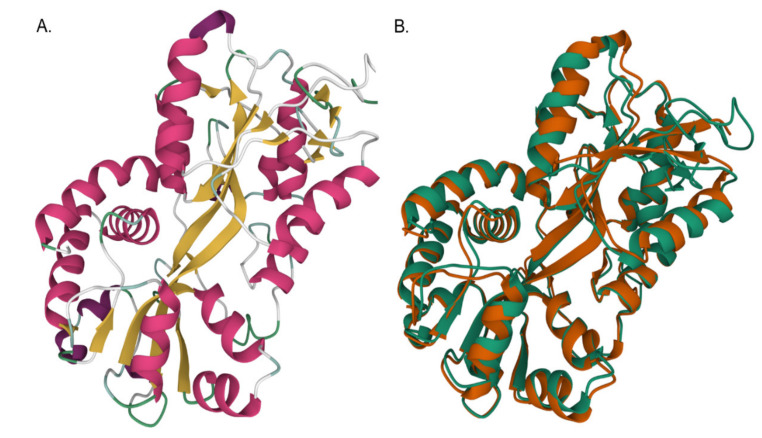
Structural model for Am069. (**A**) The best structural model for Am069 has two globular domains formed by alpha helices (pink), 3_10_-helix (magenta), coils (white), and turns (green), separated by two anti-parallel beta strands (yellow). (**B**) Structural superposition between Am069 (green) and *Synechocystis* FutA1 (brown), the protein with the highest structural similarity to Am069 (brown).

In order better define the predicted function of the *A. marginale* genes, conserved domains were identified using the National Center for Biotechnology Information (NCBI) Conserved Domain Database (CDD). On the basis of the search of this database, Am069 has a type 2 periplasmic binding fold superfamily domain. Proteins with these domains are typically involved in the uptake of various soluble substrates. However, in Am069, this conserved domain lacks predicted iron binding residues. Thus, structural models of the folded protein were generated using I-TASSER [16,17,18]. The best model for Am069 (C-score = −0.90) has two globular domains composed of alpha helices separated by two adjacent, anti-parallel beta sheets. The beta sheets act as a hinge during ligand binding [19] (Figure 6). Additionally, the proteins with the closest structural similarity to Am069, on the basis of TM-align scores, are all iron binding proteins and include Fut1A from *Synechosystis* spp. strain PCC 6803 (Protein Databank (PDB) 2PT1, TM-score = 0.890), cFbpA from *C. jejuni* (PDB 1Y4T, TM-score 0.884), and FutA from *Trichodesmium erythraeum* (PDB 6G7N, TM-score 0.876) [20,21,22]. Finally, Am069 has three conserved tyrosine residues predicted to be involved in iron coordination (Table 1, Figure S1). On the basis of these findings, we conclude there is a high likelihood that Am069 directly binds iron.

On the basis of the NCBI CDD, Am240 has the permease component typical of FbpB. Similar to FbpB, Am240 has a high degree of hydrophobicity, 12 transmembrane domains, and an ATPase-interacting loop between transmembrane regions 10 and 11. This loop is characterized by a 20 aa hydrophilic segment and has the consensus EAA-X3-G-X9-I-X-LP domain, where Xs are variable, and includes the absolutely conserved G near the middle of the domain (Figure S2) [10,23,24]. Thus, Am240 likely serves as a receptor for FbpA and forms a channel through the inner membrane for passage of molecular iron.

Finally, the ortholog of Am392 is FbpC, an ATPase that supplies energy for iron transport across the inner membrane [10,25]. The start site of Am392 was originally misannotated. On the basis of the alignments with other FbpC proteins, we corrected the start site such that Am392 was shorter by 14 aa than in the original annotation. On the basis of the NCBI CDD, Am392 has the ATPase component of ABC-type transporters. This includes the Walker A motif G-X2-G-X-GK[S/T], where Xs are variable and a Walker B motif consisting of hhhhDE, where h represents hydrophobic residues, both of which are involved in ATP binding and hydrolysis. The highly conserved linker peptide LSGGQ[Q/R/K]QR that precedes the Walker B motif and is unique to ABC transporters, and the switch region, X9-H, involved in conformational changes, are also present (Figure S3) [26,27,28].

In summary, based on a combination of structural predictions and the presence of conserved domains, Am069 likely binds and transports iron across the periplasm, Am240 retrieves iron from Am069 and transports it across the inner membrane, while Am392 is an ATPase that supplies energy for transport.

### 2.7. Differential Expression of Am069, Am240, and Am392 in Response to Altered Iron Levels

**Figure 7:**
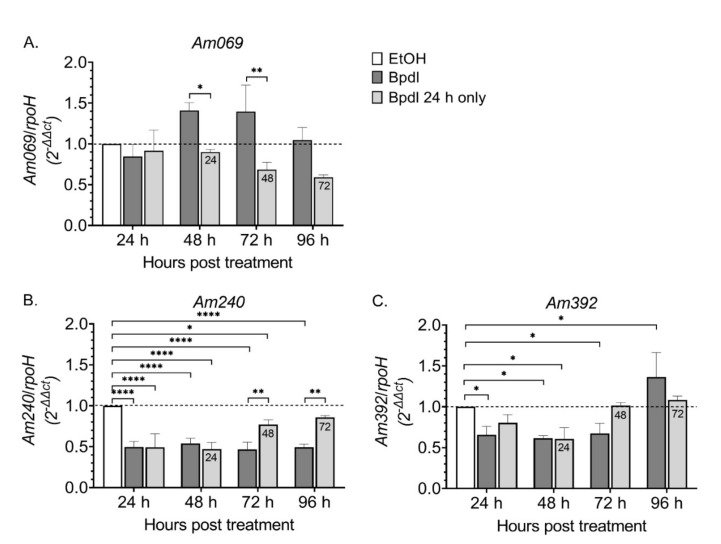
Transcriptional response of *Am069* (**A**), *Am240* (**B**), *and Am392* (**C**) to iron reduction and restoration. All cells were infected with *A. marginale* 24 h prior to the start of the experiment. Cells were treated with the carrier (EtOH), the chelator (Bpdl), or with the chelator for only 24 h (Bpdl 24 h only). The x-axis indicates time post-treatment in hours. The numbers in the light grey bars (Bpdl 24 h only) indicate the amount of time following iron restoration. The y-axis is the relative normalized expression (2^−∆∆Ct^) of the ratio of the abundance of *Am069*, *Am240*, or *Am392* transcript in Bpdl treated cells or cells treated with Bpdl for only 24 h to carrier-treated cells normalized to expression of *rpoH* under the same conditions. Error bars indicate SEM of four independent replicates for the iron depletion experiments and three independent replicates for the iron restoration experiments. Statistical significance is denoted as follows: * = *p* < 0.05, ** = *p* < 0.01, **** = *p* < 0.0001.

To provide experimental evidence suggesting *Am069*, *Am240*, and *Am392* play a role in iron transport, we measured the relative expression of these genes in response to reduction and subsequent recovery of iron levels through time (Figure 2B). There was no change in *Am069* transcript levels following 24 h of iron reduction. However, at 48 h and 72 h of iron reduction, there was a 1.4-fold increase in *Am069* transcript compared to cells treated with carrier only (Figure 7A, Table S4). In the cells that received Bpdl for only 24 h, *Am069* transcript levels trended downwards at 72 h (1.6-fold) and 96 h (1.7-fold) post treatment (48 h and 72 h following iron restoration).

*Am240* transcript was reduced twofold at all time points in response to iron reduction as compared to the cells treated with carrier only (*p* ≤ 0.0001) (Figure 7B, Table S4). In the cells that received Bpdl for only 24 h, *Am240* transcript levels partially recovered and increased 1.2-fold at 72 h and 96 h post-treatment (48 h and 72 h following iron restoration), as compared to 24 h post-treatment.

Similarly, *Am392* transcript decreased approximately 1.5-fold at 48 h and 72 h of iron reduction (*p* =< 0.05), while at 96 h, transcript levels increased 1.5-fold as compared to the cells treated with carrier only (*p* < 0.05) (Figure 7C, Table S4). In cells that received Bpdl for only 24 h, transcript levels recovered at 72 h and 96 h post-treatment (48 and 72 h following iron restoration.

## 3. Discussion

Together, our results support the hypothesis that iron is required for *A. marginale* replication in tick cells. This conclusion is based on the following: (1) live *A. marginale* as detected through transcript was readily detected, even at later time points of iron reduction; (2) *A. marginale* levels increased with the restoration of iron, indicating replication; (3) *A. marginale* colony size, according to fluorescent microscopy, remained static in the face of iron reduction, but increased with the restoration of iron, directly indicating replication. However, iron reduction also likely leads to death of some *A. marginale*. For example, between 24 h and 48 h of iron reduction, there was 1.5-fold reduction in *A. marginale* levels. The loss was more marked between 24 h and 72 h with a 2.6-fold decrease in *A. marginale* levels, although between 72 and 96 h, the levels remained stable.

Additionally, despite the lack of a *fur* regulator, *A. marginale* has some limited ability to respond to altered host cell iron levels. An important caveat is the inability to isolate the effects of iron deprivation on the host cells from the direct effects of iron deprivation on the intracellular bacteria. The approach used to deplete iron in DAE100 cells resulted in measurably decreased iron levels according to ICP/MS and a decrease in ferritin 1 protein levels in the absence of cell death [14]. Additionally, the overall low cytotoxicity of the treatment provides some assurance of the health of the cells during the experiments. However, negative effects of Bpdl and EtOH on the tick cells were measured using exclusion of trypan blue stain. In essence, this is a measure of membrane integrity. It is possible that the EtOH or Bpdl had cytotoxic effects that were not yet reflected in alteration in membrane integrity, and consequently the cytotoxic effects of EtOH and Bpdl were underestimated. For example, the decrease in *Am069* and increase in *Am392* transcripts at 96 h of iron deprivation was the opposite of what one would expect on the basis of earlier time points, suggesting some level of metabolic derangement in the cells at the later time points.

**Figure 8:**
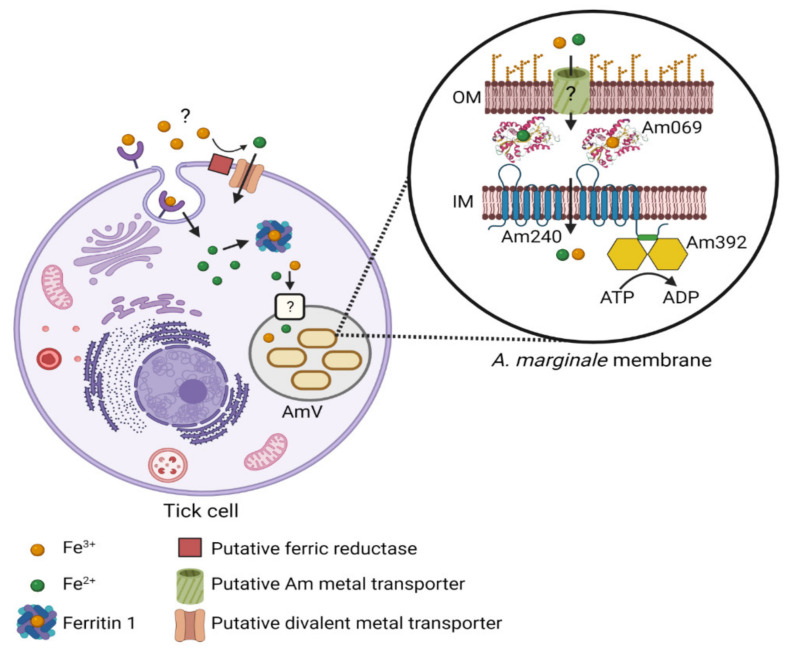
Knowledge gaps in understanding iron transport in tick cells and *A. marginale*. Molecules and mechanisms required for uptake of dietary iron in tick cells and transport of iron across the *A. marginale*-containing vacuole and *A. marginale* outer membrane are unknown and are denoted by “?” in the figure. Additionally, the requirement for Fe^2+^ vs. Fe^3+^ in iron transport in tick cells and *A. marginale* is unknown and is denoted by the use of both Fe^2+^ and Fe^3+^ in the diagram. Am069 (an FbpA ortholog) likely transports iron through the periplasm and delivers it to Am240 (FbpB ortholog), a proposed cytoplasmic permease, which transports iron across the inner membrane. Am240 (an FbpC ortholog) is an ATPase that likely interacts with Am240 and provides energy for transport.

Bacterial pathogens influence iron uptake and availability in host cells. In the case of the *Anaplasmatceae*, many knowledge gaps remain in our understanding of iron acquisition (Figure 8). Additionally, the iron uptake and transport mechanisms in the *Anaplasma* spp. differ from those of the *Ehrlichia* spp. In *E. chaffeensis*, for example, transferrin receptors accumulate on the pathogen-containing vacuole early in infection of mammalian monocytic cell lines, and the *E. chaffeensis* vacuole interacts with the TfR recycling endosome [3,29]. Decreased transcription of TfR markedly inhibits *E. chaffeensis* infection, and this effect can be reversed with the addition of holotransferrin [3,29]. Additionally, the *E. chaffeensis* vacuole is mildly acidic, thus facilitating the dissociation of iron from transferrin [29]. Together these data indicate that holotransferrin is one source of iron for *E. chaffeensis* in monocytes. In a related or additional mechanism for iron acquisition, an *E. chaffeensis* effector protein, Etf-3, is exported into the macrophage cytoplasm via the type IV secretion system (T4SS) and induces breakdown of ferritin and release of Fe^2+^ into the cytosol, thus increasing availability of iron for *E. chaffeensis* [5].

In contrast, transferrin receptors do not co-localize with *A. phagocytophilum*-containing vacuoles in monocytes [3]. Erythrocytes, the primary host cell for *A. marginale* in mammals, do not express transferrin receptors, while ticks lack identifiable transferrin receptors [14]. Additionally, the *A. marginale*-containing vacuole in *D. andersoni* cells has a neutral pH [30]. Thus, *Anaplasma* spp. likely use iron from sources other than transferrin. Additionally, *A. marginale* and *A. phagocytophilum* lack an ortholog of Eft-3, although enhancing ferritinophagy via other effectors or mechanisms is a possible means of iron acquisition in tick cells.

The final steps in iron uptake by Gram-negative bacteria are transport across the bacterial outer membrane, periplasm, and inner membranes. *Neisseria* spp. have a transferrin-binding protein, TbpA, which binds holo-transferrin and then passes the iron to nFbpA for transport across the periplasm [15,31]. While an outer membrane-exposed iron-binding protein has not been identified in the *Anaplasmataceae*, they all have orthologs of the siderophore-independent iron transporter, FbpA, FbpB, and FbpC (Figure 8). Unlike other Gram-negative bacteria that have this system, the *Anaplasmataceae*, including *A. marginale*, lack an identifiable *fur* regulator, and these three genes do not form an operon. However, transcript levels of these genes in *A. marginale* were modestly responsive to the manipulation of iron levels in tick cells, although the responses were not homogeneous among genes.

*Am069*, the predicted periplasmic, iron transport protein, was upregulated in response to iron reduction, similar to the orthologs nFbpA and hFbpA in *Neisseria* spp. and *Haemophilus* spp., respectively, as well as FutA1 of *Synechosystis* spp. [9,13,32]. This response was modest, which may reflect a reduced ability to respond to environmental changes compared to facultative bacteria, likely due to their residence in a stable intracellular niche. Similarly, the related pathogen, *Rickettsia rickettsii*, lacks a *fur* regulator, but has a limited transcriptional response to iron depletion in host cells [33].

In contrast, *Am240* and *Am392* were both downregulated in the first 24–72 h of iron reduction, although expression levels recovered once iron levels were restored, indicating some ability to adapt to altered cellular environments. The difference in transcriptional response between the periplasmic iron transporter (*Am069*), the permease (*Am240*), and the ATPase (*Am392*) may reflect their different roles in iron transport. In the face of iron reduction, the increase in *Am069* transcript may be a direct response to increased demand for iron, while the decrease in *Am240* and *Am392* may reflect a more general response to iron reduction and a resulting decrease in the ability of the bacteria to generate energy.

Interestingly, two of the three proteins with the greatest structural similarity to Am069 are FutA proteins from Cyanobacteria (*Synechocyctis* sp. and *T. erythraeum*), while the third is cFbpA from *C. jejuni* (Table 1). Unlike the canonical nFbpA and hFpbA, *Synechocystis* sp. FutA1 and *C. jejuni* cFbpA preferentially bind Fe^2+^, while *T. erythraeum* is thought to bind Fe^3+^, although there is less experimental data for this organism [20,21,22]. Cyanobacteria, as one of the oldest lifeforms on earth, evolved in low-oxygen conditions and are thought to have maintained the ability to transport Fe^2+^, which is more abundant in anaerobic conditions, but acquired ferrireductases in order to adapt to an aerobic environment [20]. *C. jejuni* has an intracellular life stage that is likely microaerophilic, which may account for the binding preference for Fe^2+^ of cFbpA. *A. marginale*, as an obligate intracellular bacterium, also likely resides in a microaerophilic environment [34]. Thus, it is possible that Am069 has an affinity for Fe^2+^, although this must be experimentally demonstrated.

*Synechocystis* Fut1A, *C. jejuni* cFbpA, and *T.* *erythraeum* FutA coordinate iron in a different fashion than the canonical nFbpA and hFbpA, which use a histidine, glutamate, two adjacent tyrosine residues, and a phosphate counter ion (Table 1, Figure S1). In contrast, on the basis of X-ray crystallography, *C. jejuni* cFbpA and *Synechocystis* sp. FutA1 use one histidine and four tyrosine residues for iron coordination (Table 1, Figure S1) [20,22]. *T. erythraeum* FutA uses three tyrosine residues, and no counter ion, although this protein maintains a conserved histidine and tyrosine residues that are involved in iron coordination in FutA1 of *Synechocystis* sp. and cFbpA of *C. jejuni* (Table 1, Figure S1) [21]. Iron coordination in *A. marginale* is predicted to be most similar to *T. erythraeum* FutA using only three tyrosine residues (Table 1, Figure S1). Unlike, *T. erythraeum* FutA, Am069 does not have the conserved histidine and tyrosine residues in the N terminus of the proteins that contribute to iron coordination in *Synechocystis* FutA1 and *C. jejuni* cFbpA (Figure S1).

In summary, we demonstrated that *A. marginale* requires iron for replication in tick cells. Based on bioinformatics and protein modeling, we found that *Am069*, *Am240*, and *Am392*, although not organized in an operon, are orthologs of the siderophore-independent iron transport system, FbpA, found in many Gram-negative bacteria (Figure 8). Although *A. marginale* lacks a *fur* regulator, these genes are differentially expressed in response to iron starvation and recovery. Additionally, FutA1 from the Cyanobacterium *Synechocystis* sp. has the greatest structural similarity to Am069. Protein localization experiments are required to determine if any portion of Am069 is surface-exposed, and thus a potential vaccine target. More broadly, X-ray crystallography, heterologous expression, and iron binding experiments must be performed to determine if Am069 is required for iron transport, the binding preference for Fe^2+^ as compared to Fe^3+^, and to determine how iron coordination is achieved. Together, this information will help build our understanding of the unique physiology of the *Anaplasmataceae*.

## 4. Methods

### 4.1. DAE100 Cell Culture Conditions and A. marginale Strain

DAE100 cells (source: Dr. Uli Munderloh in the department of Entomology at the University of Minnesota) isolated from *D. andersoni* embryonated eggs were used in all experiments [35]. Cells were maintained at 34 °C and grown in L15B complete medium as previously described [36]. The St. Maries strain of *A. marginale*, originally isolated at Washington State University and maintained by the USDA-ARS Animal Disease Research Unit at Washington State University, was used in all experiments [37]. L15B buffered with HEPES (Millipore-Sigma, Burlington, VT, USA), NaHCO_3_ (Millipore-Sigma, Burlington, VT, USA) and NaOH (Millipore-Sigma, Burlington, VT, USA) (*Anaplasma* medium) was used to maintain *A. marginale* infected DAE100 cells [38]. To ensure iron stores were adequately depleted, DAE100 cells used in iron depletion experiments were grown in L15B medium without ferrous sulfate (FeSO_4_) for two weeks prior to each experiment [14].

### 4.2. Production, Storage, and Quantitation of Host Cell-Free A. marginale

To isolate *A. marginale* free of host cells, flasks of heavily infected DAE100 cells were pelleted via centrifugation at 1800× *g* for 15 min. The cell pellet was resuspended in sucrose-phosphate-glutamate buffer (SPG) made of 230mM sucrose (Avantor, Allentown, PA, USA), 4.41 mM KH2PO4 (Avantor, Allentown, PA, USA), 6.98 Mm K2HPO4 (Millipore-Sigma, Burlington, VT, USA), and 4.92 mM C_5_H_8_KNO_4_·H2O (Millipore-Sigma, Burlington, VT, USA) dissolved in water (ThermoFisher Scientific, Fitchburg, WI, USA), vortexed, then sonicated in a cup horn sonicator (Fisherbrand Model 705 Sonic Dismembrator) at 30% amplitude until ≈90% of cells had lysed, as visualized on a wet mount slide. Cellular debris was pelleted by centrifugation at 200 rcf for 5 min. The *A. marginale* was aliquoted into cryovials and preserved at −80 °C.

*Anaplasma marginale* DNA was extracted from cryopreserved SPG stocks using a DNeasy Blood and Tissue kit (Qiagen, Valencia, CA, USA). The quantity of *A. marginale* per microliter of SPG stock was calculated using quantitative PCR and a standard curve with *msp5*-specific primers and PerfeCTa SYBR Green FastMix (Quantabio, Beverly, MA, USA) [39]. As *msp5* is a single copy gene, the number of msp5 copies reflects the number of bacteria (Table 2).

### 4.3. Experimental Design Overview

Cells were infected with *A. marginale* 24 h prior to the initiation of treatments, except for the experiments to determine the cytotoxic effects of Bpdl and EtOH on DAE100 cells. To induce iron depletion in all experiments, DAE100 cells were treated every 24 h up to 96 h with Bpdl (Figure 2A). To determine if the effects of iron depletion were reversible, *A. marginale*-infected DAE100 cells were treated with Bpdl for 24 h only. Following this treatment, cells then received iron-replete *Anaplasma* media for the reminder of the experiment (Figure 2B). To confirm that the reduction in *A. marginale* levels was due to iron reduction in the host cell rather than unknown effects of the chelator, *A. marginale*-infected cells were treated every 24 h with Bpdl plus various concentrations of FeSO_4_ (Figure 2A). Cells treated with the carrier only, EtOH, served as the control.

Cells were harvested starting 24 h following the first treatment and each 24 h thereafter. Cells treated with Bpdl or the carrier were harvested up to 96 h post-treatment, while cells treated with Bpdl plus FeSO_4_, were harvested up to 72 h post-treatment. Following harvest, cells were stained with trypan blue to determine viability, or total RNA was extracted to enumerate *A. marginale* levels relative to DAE100 cells or *Am069*, *Am240*, and *Am392* transcript levels relative to the housekeeping gene *rpoH*, using RT-qPCR and calculation of the 2^−∆∆Ct^.

### 4.4. Iron Depletion and Infection of DAE100 Cells

For cytotoxicity, iron deprivation and reversal of iron deprivation studies, cellStar T25 flasks (Greiner Bio-One, Monroe, NC, USA) were seeded with 5 mL of medium containing 5 × 10^5^ cells/mL. DAE100 cells were infected at an *A. marginale* multiplicity of infection (MOI) of 350:1. For the rescue of *A. marginale* with the addition of FeSO_4_ in the presence of Bpdl, 24-well CellStar cell culture plates (Greiner Bio-One, Monroe, NC, USA) were seeded with 5 × 10^5^ of DAE100 cells in 500 µL of medium. Because of the smaller area of the wells as compared to T25 flasks, an MOI of 100:1 was used.

To infect DAE-100 cells, vials of cell-free *A. marginale* cryopreserved in SPG buffer were thawed and pelleted by centrifugation at 13,000× *g* for 5 min. The *A. marginale* pellets were resuspended in either buffered L15B complete medium or in L15B complete medium without FeSO_4_. The infected flasks or plates were spun at 200× *g* for 5 min to force contact between the cells and bacteria, then incubated at 34 °C for 2 h to allow pathogen entry. After 2 h, the medium was replaced with *Anaplasma* medium using gentle pipetting, and the cells were incubated for 24 h. Plates were incubated in a sealed BD Campy Container with a GasPak (Scientific Equipment Company, Aston, PA USA).

At 24 h post-infection, cells were fed with regular *Anaplasma* medium, *Anaplasma* medium containing 100 µM of Bpdl (Sigma Aldrich, St. Louis, MO, USA), or *Anaplasma* medium containing 2 µL/mL of 100% of EtOH (Koptec, King of Prussia, PA, USA), as the carrier control for Bpdl. Treatments continued every 24 h for 96 h. At each time point, 24 h, 48 h, 72 h, and 96 h post-treatment, cells were pelleted and frozen in RNAlater (Ambion, Austin, TX, USA) at −80 °C for RNA extraction (Figure 1A). These experiments were performed four independent times.

### 4.5. Cytotoxicity of Iron Chelator on DAE100 Cells

Trypan blue, which only stains non-viable cells, was used to measure the cytotoxicity of 100 µM of Bpdl and EtOH on uninfected and *A. marginale*-infected DAE100 cells. Cells were harvested every 24 h starting 24 h after treatment with Bpdl or EtOH for 96 h. For staining, cells were mixed 1:1 with 0.4% trypan blue (Millipore-Sigma, Burlington, VT, USA), and viable and non-viable cells were counted using a hemocytometer and light microscopy. Three technical replicates were counted in each experiment. The experiment was performed three independent times.

### 4.6. Reversal A. marginale Growth Inhibition

To determine if the effect of iron depletion on *A. marginale* replication was reversible, infected DAE100 cells were treated with 100 µM Bpdl for 24 h. Cells then received *Anaplasma* medium for the remainder of the experiment. Cells were treated and harvested every 24 h up to 96 h and stored for RNA extraction as described above (Figure 1B). These experiments were performed three independent times.

### 4.7. Rescue of A. marginale Replication by Addition of FeSO_4_

In order to restore iron availability in the presence of 100 µM Bpdl, *A. marginale*-infected DAE100 cells were treated daily for 24 h, 48 h, and 72 h with 20 mM, 50 mM, or 100 mM FeSO_4_ (Millipore-Sigma, Burlington, VT, USA). Controls groups were treated with either 100% EtOH at 2 µL/mL or 100 µM Bpdl on the same schedule. These experiments were performed four independent times.

### 4.8. RT-qPCR

Specific primers were designed to amplify *rpoH*, *Am069*, *Am240*, and *Am392* of *A. marginale* and *β-actin* of *D. andersoni* using Integrated DNA Technologies (IDT) and were tested for amplification efficiency (Table 2). RNA was extracted from DAE100 cells using an RNeasy Mini Kit (Qiagen, Valencia, CA, USA) following the manufacturer’s instructions. Extracted RNA was treated with DNAse twice using a Turbo DNase Kit (Ambion, Austin, TX, USA) and cleaned using an RNA Clean and Concentrator-25 kit (Zymoresearch, Irvine, CA, USA). RNA concentrations were measured using a Nanodrop ND-1000 spectrophotometer (ThermoFisher Scientific, Fitchburg, WI, USA). RNA was converted to cDNA using the Superscript III Reverse Transcriptase kit (Invitrogen, Carlsbad, CA, USA) as described in the manufacturer’s protocol.

Next, a CFX96 Touch Real-Time PCR Detection System (Bio-Rad, Hercules, CA, USA) was used to perform RT-qPCR. Each RT-qPCR reaction contained cDNA, SsoAdvanced™ Universal SYBR Green Supermix (Bio-Rad, Hercules, CA, USA), RNase/DNase free water, and 20 nM forward and reverse primers (Integrated DNA Technologies, Coralville, IA, USA). Thermocycling conditions for all reactions were as follows: denaturation at 95 °C for 30 s, then 40 cycles of 95 °C for 5 s, 60 °C for 15 s, followed by a melt curve analysis from 65–95 °C in 0.5° increments for 5 s/step.

2^−∆∆CT^ was used to quantify *A. marginale* relative to DAE100 cells. Specifically, the ratio of the abundance of *A. marginale rpoH* transcript in treated cells (Bpdl, Bpdl-24 h only, and Bpdl + FeSO_4_) to EtOH-only treated cells (at the same time post infection) was normalized to *D. andersoni β-actin* under the same treatment conditions. Using *D. andersoni β-actin* compensates for any differences in the number of DAE100 cells. The same approach was used to measure the relative transcript levels of *Am069*, *Am240*, and *Am392*. In this case, the ratio of the abundance of the target gene (*Am069*, *Am240*, or *Am392*) transcript in treated cells (Bpdl or Bpdl-24 h only) to the target gene in EtOH-only treated cells (at the same amount of time post *A. marginale* infection) was normalized to expression of the housekeeping gene, *rpoH*, under the same conditions. The use of *rpoH* compensates for differences in *A. marginale* levels between samples.

### 4.9. Immunofluorescent Microscopy and Image Analysis

To visually evaluate the effects of iron reduction on *A. marginale* growth, 1 × 10^6^ DAE100 cells were seeded in a Nunc™ Lab-Tek™ II Chamber Slide™ System (ThermoFisher Scientific, Fitchburg, WI, USA). Cells were infected with *A. marginale* using an MOI of 350:1, spun at 200× *g* for 5 min, then incubated for 2 h before replacing the media via gentle pipetting. Cells were treated daily with Bpdl or EtOH, as described above (Figure 1). At each time point, slides were fixed with 4% PFA (Invitrogen, Carlsbad, CA, USA) for 10 min, then rinsed well with phosphate buffered saline PBS (Quality Biological, Gaithersburg, MD, USA). All antibodies were diluted to 0.5 µg/mL in PBS with 0.05%, Tween 20 (Thermo Scientific Pierce, Fitchburg, WI, USA) and 3.0% bovine serum albumin (Millipore-Sigma, Burlington, VT, USA). *Anaplasma marginale* outer membranes were labeled for 1 h with AnaR49A1 anti-Msp2 primary antibody [30] (source: Guy H. Palmer, in the Paul Allen School for Global Health at Washington State University), rinsed, then labeled with Goat anti-Mouse IgG (H+L) Alexa Fluor 594 secondary antibody (Invitrogen, Carlsbad, CA, USA) and Alexa Fluor 488 Phalloidin (Invitrogen, Carlsbad, CA, USA) for 1 h. Slides were rinsed and mounted with Prolong Diamond Antifade with DAPI (Invitrogen, Carlsbad, CA, USA) and coverslips.

Deconvolution microscopy was performed using an Axio Imager.M1 microscope (Carl Zeiss Microimaging, Thornwood, NY, USA) equipped with an X-Cite 120 Fl Illuminating system (EXFO Photonic Solutions, Mississauga, ON, Canada) and an AxioCam MR3 digital camera. Image acquisition was performed under two settings. To estimate bacterial load (Alexa Fluor 594) and cellularity (Alexa Fluor 488) for each treatment at each time point, six non-adjacent regions were blindly selected and imaged as 512 × 512 pixel two-channel image stacks using a Plan-Apochromat 20×/0.8 M7 objective (pixel scaling: 0.324 nm × 0.324 nm) at z-axis steps of 250 nm. Examples of bacterial colony morphology (Alexa Fluor 594) were then acquired as 256 × 256 pixel single-channel image stacks using a Plan-Apochromat 63×/1.4 oil M7 objective (pixel scaling: 0.103 × 0.103 nm) and 200 nm z-axis steps. Treatment with Bpdl reduced DAE100 cell adherence to slides in a time-dependent manner. Thus, only four of six image stacks were suitable for measurement of the ‘Bpdl 24 h only’ treatment group at 72 h, and none of the image stacks for either Bpdl treatment group were suitable for measurement at 96 h. All image processing and measurements were done using the Fiji distribution of ImageJ software (http://fiji.sc/ accessed on 23 March 2022) [40].

Deconvolution of original image stacks was accomplished using the DeconvolutionLab2 plugin [41]. For image stacks acquired using the 20× objective, the Richardson–Lucy Total Variation algorithm (lambda 0.001) was used with z-axis Tukey apodization and a residual less than 0.01 as a final iteration constraint. For 63× objective acquisitions, the Richardson–Lucy algorithm was used with no apodization and a residual less than 0.001 as a final iteration constraint. Theoretical point spread functions were generated using the Richards and Wolf 3D optical model as implemented in the PSF Generator plugin [42].

The ‘auto local threshold’ function was used to objectively segment fluorescent structures, using the image stack histogram and default settings of the Bernsen algorithm for Alexa Fluor 488, and the Phansalkar algorithm for Alexa Fluor 594. The ‘analyze particles’ function was used to measure the area (sq microns) of each discrete threshold structure present in each image stack slice. This experiment was performed once.

### 4.10. In Silico Identification of A. marginale Genes Involved in Iron Transport

To identify *A. marginale* genes potentially involved in iron transport, we first used the KEGG to search for orthologs of ABC transporters that bind and transport iron. The three resulting genes, *Am069*, *Am240*, and *Am392*, were translated in silico, and the CDD from NCBI was used to search for conserved domains.

On the basis of KEGG orthology, Am069 is predicted to bind iron, but this was not supported by the presence of conserved domains. To help address this discordance, we performed structural modeling using I-TASSER [16,17,18]. In contrast, Am240 and Am392 both have conserved domains that give a strong indication of function, and thus structural modeling for these two proteins was unnecessary.

For structural modeling of Am069, the amino acid sequence was uploaded into I-TASSER. The resulting best structural model among the five provided by I-TASSER is reported in the results. Additionally, the three proteins in the PDB with the closest structural similarity to Am069, on the basis of TM-align scores, as provided by I-TASSER, are also reported. Finally, the overlay between the predicted structure of Am069 and best PDB match are presented (Figure 6).

For Figures S1–S3, multiple sequence alignments were performed with SnapGene (GSL Biotech LLC, San Diego, CA, USA) using the Clustal Omega alignment algorithm and the Clustal X coloring scheme. TMHMM was used to predict the transmembrane domains for Am 240 and its orthologs, as presented in Figure S2 [43].

### 4.11. Statistical Analysis

GraphPad Prism V9 Software (San Diego, CA, USA) was used for most of the statistical analyses. The data from the cytotoxicity assays and transcriptional analysis of *Am069*, *Am240*, and *Am392* were analyzed by two-way ANOVA followed by a Dunnett’s test for multiple comparisons. Raw numbers from the cytotoxicity assays were used in the statistical analysis. The data from RT-qPCR experiments quantitating *A. marginale* relative to tick cell numbers were log2 transformed, and significant differences between groups were determined using two-way ANOVA followed by a Tukey’s test for multiple comparisons.

Intracellular growth of bacterial colonies was measured as the total area of threshold anti-Msp2 fluorescence divided by the total area of threshold phalloidin. The effects of treatment, incubation period, and the interaction term were analyzed using a generalized linear model (PROC GLIMMIX; SAS version 9.4, SAS Institute Inc., Cary, NC, USA). The data were well fit by the beta distribution, and comparisons of interest were made by including an LSMEANS statement for the interaction term sliced by the incubation periods and significance values adjusted by the step-down Bonferroni (Holm) procedure. The effects of treatments and time on colony sizes were compared by visual inspection of empirical cumulative distribution functions (PROC NPAR1WAY, EDF; SAS).

## Figures and Tables

**Table 1:** Proteins with the best structural similarity to Am069 and their iron-coordinating amino acids.

PDB	Organism	Protein	Amino Acids Involved in Iron Coordination						Counter Anion	Iron State ^a^
			HIS	GLU	TYR	TYR	TYR	TYR		
N/A	*A. marginale*	Am069	K39 ^b^	A89 ^b^	E40 ^b^	**Y170** ^c^	**Y226** ^c^	**Y227** ^c^	unk	unk
2PT1 ^d^	*Synechocystis*	FutA1	**H54**	V102 ^b^	**Y55**	**Y185**	**Y241**	**Y242**	none	Fe^2+^
1Y4T ^d^	*C. jejuni*	cFbpA	**H27**	A75 ^b^	**Y28**	**Y159**	**Y215**	**Y216**	none	Fe^2+^
6G7N ^d^	*T. erythraeum*	FutA	H10 ^b^	V58 ^b^	Y11 ^b^	**Y141**	**Y197**	**T198**	none	Fe^3+^
1D9VA ^e^	*H. influenzae*	hFbpA	**H32**	**E80**	K33 ^b^	F165 ^b^	**Y218**	**Y219**	PO^4+^	Fe^3+^

Bold indicates amino acids involved in iron coordination. ^a^ Preferred state of iron for binding and transport by periplasmic transport protein. ^b^ Amino acids that do not coordinate iron or not predicted to coordinate iron but align with amino acids involved in iron coordination in other species. ^c^ Amino acids predicted to be involved in iron coordination in Am069. ^d^ Three most structurally similar proteins to Am069 on the basis of the I-TASSER structural models and TM-align scores. ^e^ The best Gene Ontology match for Am069 in PDB according to the I-TASSER structural models.

**Table 2:** Oligonucleotides used for RT-qPCR.

Gene Name	Forward Oligonucleotide (5′-3′)	Reverse Oligonucleotide (5′-3′)	Product Size (bp)
*Msp5*	CTTCCGAAGTTGTAAGTGAGGGCA	CTTATCGGCATGGTCGCCTAGTTT	203
*Am069*	AACCTCATACTGGCGAAGAAG	CTTTGTGAGCCCAAACCAATATC	117
*Am240*	GCTGGTACTGATGGAAGTGATAG	CCAGTATGCACGCAGAGTATT	125
*Am392*	GTGATACGTGAGGGCAGAATAG	TCACACACAACGGACTCAAA	122
*RpoH*	TACGTCGCAAGCCTGAAGCC	GCGGATGCCCATAGGTTGGT	170
*ß-actin*	CGCCTCCTCCTCTTCTCTG	TGTAGGTGGTCTCGTGGATG	148

## Data Availability

The data presented in this study are available as Tables S1–S4.

## Supplementary Materials

The following supporting information can be downloaded at https://www.mdpi.com/article/10.3390/ijms23073941/s1.
