# Supplementary material for: Iron Reduction in Dermacentor andersoni Tick Cells Inhibits Anaplasma marginale Replication
Source: Int J Mol Sci. 2022 Apr 1;23(7):3941. doi: 10.3390/ijms23073941 (PMC8999750; doi:10.3390/ijms23073941)
Supplement: Supplementary file 1 [file ijms-23-03941-s001.zip › Fig. S1 Am069 alignment.pdf]

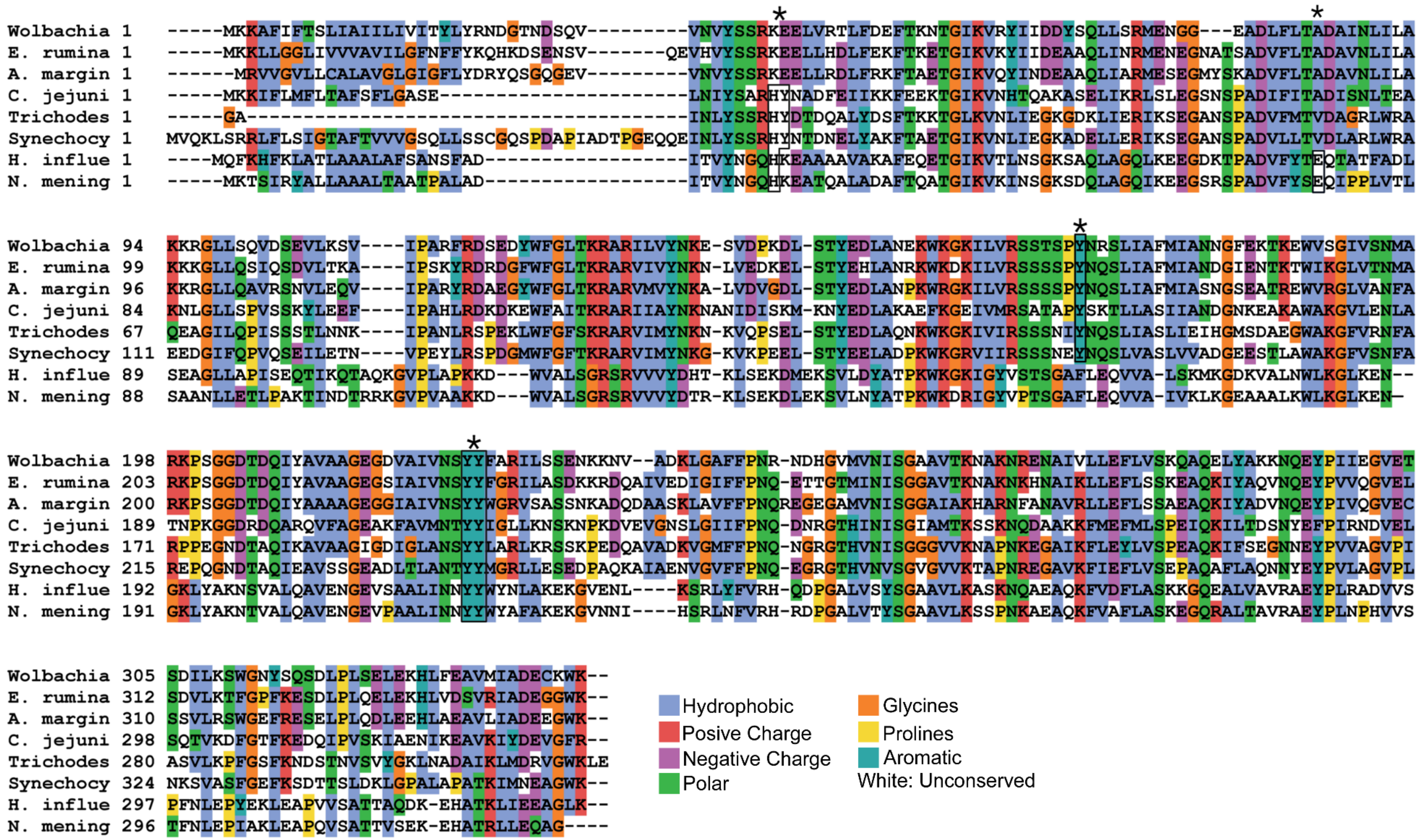

Figure S1. Amino acid alignment of *Anaplasma marginale* Am069 and FbpA/FutA/FutA1 orthologs. FbpA/FutA/FutA1 are type 2 periplasmic binding proteins that transport iron. Boxes marked by asterisks indicate conserved amino acids that coordinate or are predicted to coordinate iron binding in different organisms. NCBI or PDB accession numbers for proteins included in the alignment are as follows: Wolbachia WP_010082338.1, *Ehrlichia ruminantium* WP_158406301.1, *A. marginale* AAV86234.1, *Campylobacter jejuni* (cFbpA) QBK06856.1, *Trichodesmium erythraeum* (FutA) PDB 6G7Q_A, *Synechosystis* spp. strain PCC 6803 (FutA1) BAA16842.1, *Haemophilus influenzae* (hFbpA) WP_105881770.1, *Neisseria meningitidis* (nFbpA) MBJ7835608.1.
