# Supplementary material for: Iron Reduction in Dermacentor andersoni Tick Cells Inhibits Anaplasma marginale Replication
Source: Int J Mol Sci. 2022 Apr 1;23(7):3941. doi: 10.3390/ijms23073941 (PMC8999750; doi:10.3390/ijms23073941)
Supplement: Supplementary file 1 [file ijms-23-03941-s001.zip › Fig. S2 Am240 alignment.pdf]

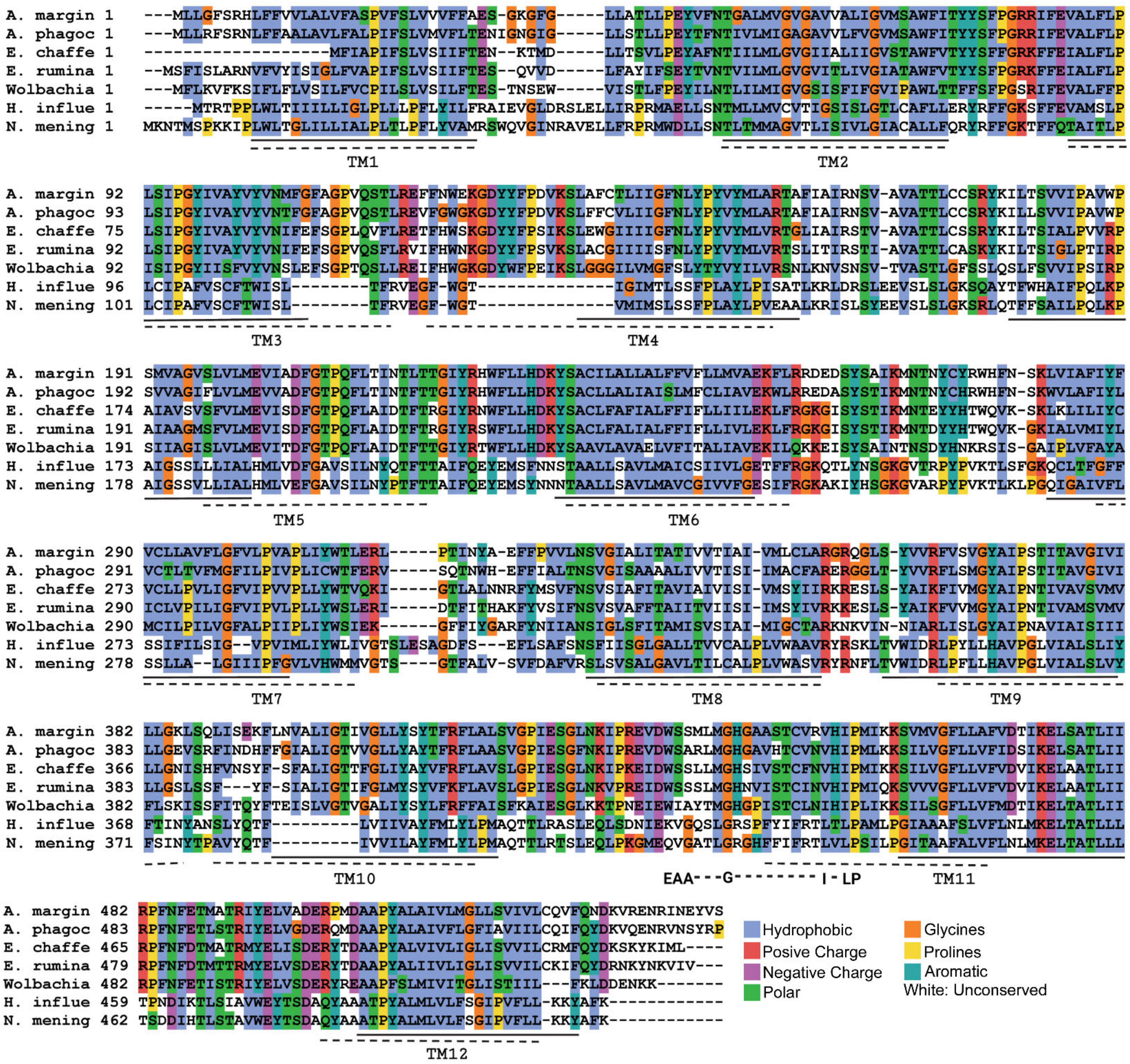
A. margin
A. phagoc
E. chaffe
E. rumina
Wolbachia
H. influe
N. mening
TM1
TM2
TM3
TM4
TM5
TM6
TM7
TM8
TM9
TM10
TM11
TM12
EAA---G--------I-LP
Hydrophobic
Posive Charge
Negative Charge
Polar
Glycines
Prolines
Aromatic
White: Unconserved

Figure S2. Amino acid alignment of *Anaplasma marginale* Am240 and FbpB orthologs. Am240 is likely a cytoplasmic permease which forms a channel for molecular iron through the inner membrane and has 12 transmembrane domains and an ATPase-interaction loop with the EAA-X3-G-X9-I-LP domain, where Xs are variable. The solid lines indicate the location of transmembrane (TM) domains for the *Anaplasmataceae*, while the dotted lines indicate the TM domains for *Haemophilus influenzae* and *Neisseria meningiditis*, based on TMHMM predictions. NCBI accession numbers for proteins included in the alignment are as follows: *A. marginale* WP_011114176.1, *A. phagocytophilum* WP_044105307.1, *Ehrlichia chaffeensis* WP_006010377.1, *E. ruminantium* WP_180811780.1, Wolbachia endosymbiont of *Brugia malayi* WP_011256241.1, *Haemophilus influenzae* WP_048953704.1, and *Neisseria meningitidis* SPY03564.1.
