# Supplementary material for: Iron Reduction in Dermacentor andersoni Tick Cells Inhibits Anaplasma marginale Replication
Source: Int J Mol Sci. 2022 Apr 1;23(7):3941. doi: 10.3390/ijms23073941 (PMC8999750; doi:10.3390/ijms23073941)
Supplement: Supplementary file 1 [file ijms-23-03941-s001.zip › Fig. S3 Am392 alignment.pdf]

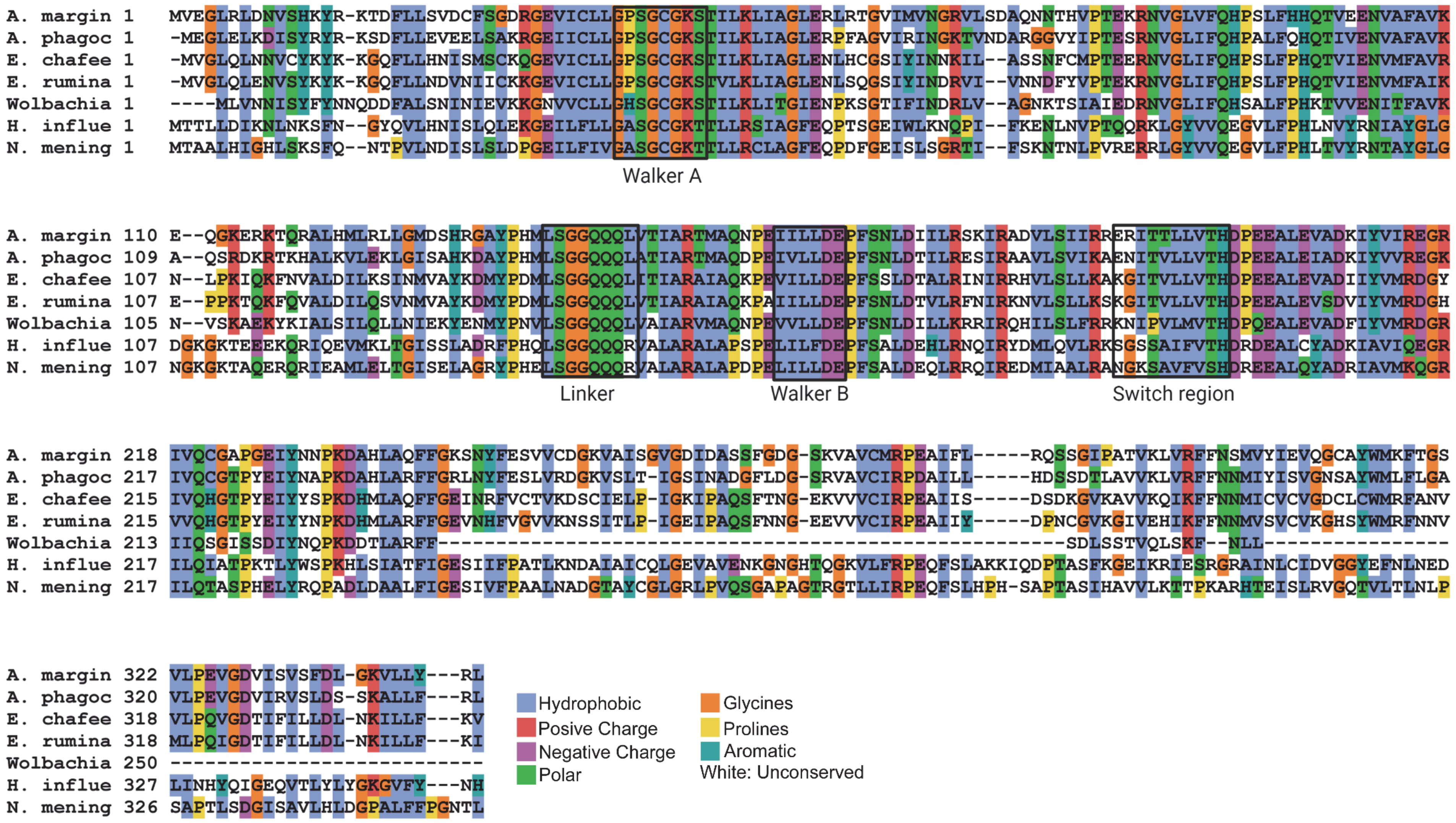

Figure S3. Amino acid alignment of *Anaplasma marginale* Am392 and orthologs. Am392 is predicted to be an ATPase that supplies energy for iron transport across the inner membrane. The conserved features that define ATPases are in outlined boxes and include: (1) Walker A (G-X2-G-X- GK[S/T]) and (2) Walker B motifs (hhhhDE, where h represents hydrophobic residues), involved in ATP binding and hydrolysis, (3) the linker peptide (LSGGQ[Q/R/K]QR), which is highly conserved and unique among the ABC transport family, and (4) the switch region (X9-H), involved in conformational changes that accompany ATP hydrolysis. Xs represent variable amino acids. NCBI accession numbers for proteins included in the alignment are as follows: *A. marginale* AAV86453.1, *A. phagocytophilum* SCV64052.1, *Ehrlichia chaffeensis* WP_011452848.1, *E. ruminantium* WP_158406426.1, Wolbachia endosymbiont of *Ctenocephalides felis* wCfeT WP_168464365.1, *Haemophilus influenzae* MBF1223503.1, and *Neisseria meningitidis* WP_118834873.1.
